# RETRACTED: Alhakamy et al. Chitosan-Based Microparticles Enhance Ellagic Acid’s Colon Targeting and Proapoptotic Activity. *Pharmaceutics* 2020, *12*, 652

**DOI:** 10.3390/pharmaceutics16020196

**Published:** 2024-01-30

**Authors:** Nabil A. Alhakamy, Osama A. A. Ahmed, Mallesh Kurakula, Giuseppe Caruso, Filippo Caraci, Hani Z. Asfour, Anas Alfarsi, Basma G. Eid, Amir I. Mohamed, Nabil K. Alruwaili, Wesam H. Abdulaal, Usama A. Fahmy, Hani A. Alhadrami, Basmah M. Eldakhakhny, Ashraf B. Abdel-Naim

**Affiliations:** 1 Department of Pharmaceutics, Faculty of Pharmacy, King Abdulaziz University, Jeddah 21589, Saudi Arabia; nalhakamy@kau.edu.sa (N.A.A.); oaahmed@kau.edu.sa (O.A.A.A.); mr_alfarsi8@hotmail.com (A.A.); uahmedkauedu.sa@kau.edu.sa (U.A.F.); 2 Advanced Drug Delivery Research Group, Faculty of Pharmacy, King Abdulaziz University, Jeddah 21589, Saudi Arabia; 3 Center of Excellence for Drug Research and Pharmaceutical Industries, King Abdulaziz University, Jeddah 21589, Saudi Arabia; 4 Department of Biomedical Engineering, University of Memphis, Memphis, TN 38152, USA; mkrakula@memphis.edu; 5 Oasi Research Institute—IRCCS, Via Conte Ruggero, 73, 94018 Troina, Italy; forgiuseppecaruso@gmail.com (G.C.); fcaraci@unict.it (F.C.); 6 Department of Drug Sciences, University of Catania, 95125 Catania, Italy; 7 Department of Medical Microbiology and Parasitology, Faculty of Medicine, King Abdulaziz University, Jeddah 21589, Saudi Arabia; hasfour@kau.edu.sa; 8 Department of Pharmacology and Toxicology, Faculty of Pharmacy, King Abdulaziz University, Jeddah 21589, Saudi Arabia; beid@kau.edu.sa; 9 Department of Pharmaceutics and Industrial Pharmacy, Military Medical Academy, Cairo 11757, Egypt; miroami@gmail.com; 10 Department of Pharmaceutics, Faculty of Pharmacy, Jouf University, Skaka P.O. Box 2014, Saudi Arabia; nabilalruwaili@gmail.com; 11 Department of Biochemistry, Cancer Metabolism and Epigenetic Unit, Faculty of Science, King Abdulaziz University, Jeddah 21589, Saudi Arabia; whabdulaal@kau.edu.sa; 12 Department of Medical Laboratory Technology, Faculty of Applied Medical Sciences, King Abdulaziz University, P.O. Box 80402, Jeddah 21589, Saudi Arabia; hanialhadrami@kau.edu.sa; 13 Special Infectious Agent Unit (Biosafety Level 3), King Fahd Medical Research Centre, P.O. Box 80402, Jeddah 21589, Saudi Arabia; 14 Department of Clinical Biochemistry, Faculty of Medicine, King Abdulaziz University, Jeddah 21589, Saudi Arabia; beldakhakhny@kau.edu.sa

The journal retracts the article “Chitosan-Based Microparticles Enhance Ellagic Acid’s Colon Targeting and Proapoptotic Activity” [1], cited above. 

Following publication, concerns were brought to the attention of the publisher regarding overlap of the images.

Adhering to our complaints procedure, an investigation was conducted that confirmed the overlap of Figure 7 with a figure in a previously published article [2], listing different experimental time points [2]. Furthermore, an overlap between Figures 4 and 5 within this article [1] was also confirmed. 

While the authors fully cooperated with the Editorial Office during the investigation, they were unable to satisfactorily explain the overlap of the figures, nor could they meet the required quality standards for raw images in order to consider a correction as per the journal’s original image requirements policy (https://www.mdpi.com/journal/pharmaceutics/instruc-tions#oriimages). As a result, the Editorial Board and Editor-in-Chief were unable to confirm the reliability of the findings and subsequently decided to retract this paper.

This retraction was approved by the Editor-in-Chief of the journal *Pharmaceutics*.

The authors did not agree to this retraction.
